# Genetic improvement of Egyptian cotton (*Gossypium barbadense* L.) for high yield and fiber quality properties under semi arid conditions

**DOI:** 10.1038/s41598-024-57676-w

**Published:** 2024-04-02

**Authors:** Sobhi F. Lamlom, W. M. B. Yehia, H. M. K. Kotb, Ahmed M. Abdelghany, Adnan Noor Shah, Ehab A. A. Salama, Mohamed M. A. Abdelhamid, Nader R. Abdelsalam

**Affiliations:** 1 Plant Production Department, Faculty of Agriculture (Saba Basha), Alexandria University, Alexandria, 21531 Egypt; 2 Cotton Breeding Department, Cotton Research Institute, Agriculture Research Center, Giza, Egypt; 3 Crop Science Department, Faculty of Agriculture, Damanhour University, Damanhour, 22516 Egypt; 4 Department of Agricultural Engineering, Khwaja Fareed University of Engineering and Information Technology, Rahim Yar Khan, 64200 Punjab Pakistan; 5 Agricultural Botany Department, Faculty of Agriculture (Saba Basha), Alexandria University, Alexandria, 21531 Egypt

**Keywords:** Cotton (*Gossypium barbadense*), Genetic improvement, Pedigree selection, Yield, Fiber quality, Semiarid conditions, Genetics, Environmental sciences

## Abstract

Between 2016 and 2018, the Agriculture Research Center's Sakha Agriculture Research Station conducted two rounds of pedigree selection on a segregating population of cotton (*Gossypium barbadense* L.) using the F_2_, F_3_, and F_4_ generations resulting from crossing Giza 94 and Suvin. In 2016, the top 5% of plants from the F_2_ population were selected based on specific criteria. The superior families from the F_3_ generation were then selected to produce the F_4_ families in 2017, which were grown in the 2018 summer season in single plant progeny rows and bulk experiments with a randomized complete block design of three replications. Over time, most traits showed increased mean values in the population, with the F_2_ generation having higher Genotypic Coefficient of Variance (GCV) and Phenotypic Coefficient of Variance (PCV) values compared to the succeeding generations for the studied traits. The magnitude of GCV and PCV in the F_3_ and F_4_ generations was similar, indicating that genotype had played a greater role than the environment. Moreover, the mean values of heritability in the broad sense increased from generation to generation. Selection criteria I2, I4, and I5 were effective in improving most of the yield and its component traits, while selection criterion I1 was efficient in improving earliness traits. Most of the yield and its component traits showed a positive and significant correlation with each other, highlighting their importance in cotton yield. This suggests that selecting to improveone or more of these traits would improve the others. Families number 9, 13, 19, 20, and 21 were the best genotypes for relevant yield characters, surpassing the better parent, check variety, and giving the best values for most characters. Therefore, the breeder could continue to use these families in further generations as breeding genotypes to develop varieties with high yields and its components.

## Introduction

Cotton is considered the most important fiber crop in the world, with Egyptian cotton (*Gossypium barbadense* L.) being renowned as the best fiber crop globally and remaining significant crops in Egypt^1–4^. Cotton breeders worldwide aim to enhance early maturity, high yield, and high-quality cotton varieties. However, selecting for high yield in cotton breeding is challenging due to its complexity and susceptibility to environmental and genetic factors. Direct selection based solely on yield is difficult and inefficient^5–7^, because of genotype x environment interactions, low heritability, polygenic nature, and non-additive gene action^8^. To address these challenges, the selection index technique was proposed to simultaneously improve multiple traits simultaneously, achieving superior results compared to direct selection. This method effectively select elite genotypes what superior yield and fiber traits^9–13^. In contrast, the pedigree selection method may less effective in identifying superior genotypes^14^. Timely maturity, known as earliness, cotton cultivation is crucial to prevent damage from frost, insects, diseases, conserve soil moisture and prevent weathering of open cotton. Cotton can also be used in a multi-crop system^15^,particularly in Egypt, where, it is rotated with winter crops like wheat. Studies have shown that a rotation system of wheat and cotton can be effective^16^. Early maturity offers various benefits, including better development in favorable weather, fitting into a double-cropping pattern with minimal impact on yield, higher water use efficiency in irrigated areas, and reduced damage from boll worms^17^. Although various selection methods have been used to enhance cotton traits, pedigree selection is the most popular among plant breeders due to its versatility, speed, and compatibility with genetic studies^18^. Most Egyptian cotton varieties were produced using pedigree selection. Studies have shown decreased Genotypic Coefficient of Variance (GCV) and Phenotypic Coefficient of Variance (PCV) after two cycles of pedigree selection^17^. Researchers have reported significant genotypic differences for various cotton traits in different generations and strains, with high to moderate broad-sense heritability estimates for all traits in direct selection for yield and its components. Broadsense heritability estimates for seed cotton yield/plant, boll weight, and number of bolls/plant have been reported as 0.98, 0.96, and 0.96, respectively^16,19,20^.

Directly selecting cotton plants based on yield alone is a challenging task due to the complex nature of the trait. Yield is greatly influenced by environmental conditions, as well as genotype x environment interactions, which diminishes the effectiveness of using yield as the only selection criterion^11,21,22^. Moreover, other factors like the polygenic nature of the trait, low heritability, linkage, and non-additive gene action may hinder the effectiveness of selection, especially in the early segregating generations. To overcome these challenges, the selection index method is proposed as a more efficient alternative approach to simultaneously improve multiple traits that are more heritable and correlated^4,23–26^. The selection index method has been shown to be more efficient than pedigree selection in isolating superior elite families in most studied traits. , increasing the probability of identifying desirable genotypes. However, the reliability of the parameter estimates used in constructing the selection index is crucial for its efficiency and may be subject to large sampling errors, including biases due to genotype by environment interaction^27–30^. To improve the efficiency of selection, it is necessary to consider heritability estimates simultaneously with genetic advances expressed as a percentage of the mean. Understanding heritability helps plant breeders predict the behavior of succeeding generations, make desirable selections, and assess the magnitude of genetic improvement through selection. Several studies have reported genetic variability for yield traits in cotton^31–34^. However, the estimates of broad-sense heritability for the characters studied were low to moderate, indicating that heritability alone is not a sufficient indication of the genetic progress that can be achieved through selection. Therefore, other traits and selection criteria should be considered when breeding cotton plants to improve yield and other desirable characteristics^16,17,35–38^. The primary objective of this research was to (i) identify cotton genotypes suited to semi-arid climates that produce high yields and fiber quality meeting textile industry standards, and (ii) evaluate predicted and actual improvements in key economic traits through the application of different selection indices during the breeding process.

## Materials and methods

### Plant materials

The study was conducted at Sakha Agricultural Research Station for three consecutive growing seasons (2017, 2018, and 2019). The materials used for the study were intraspecific cotton (*Gossypium barbadense* L.) cross (Giza 94 × Suvin) from the F_2_, F_3_, and F_4_ generations. Giza 94 is known for its high yield, high lint percentage, and earliness, while Suvin is known for its high yield and earliness. The F_1_ generation was self-pollinated, and the resulting seeds were used for the F_2_ generation. In the 2017 season, the F_2_ population and their parents were grown with a spacing of 70 cm between rows and plants, and one plant per hill was maintained. The plants were self-pollinated by covering the flowers with craft paper bags, and 375 selected plants were harvested separately. The plants were evaluated based on the first opening flower, boll weight, seed cotton yield, lint percentage, and lint index, with a selection intensity of 5%. The plants with the highest performance for each criterion were maintained, resulting in 65 selected progenies for the F_3_ generation. In the 2017 season, 65 self-selected plants from the F_2_ generation were used to raise the F_3_ generation. The F_3_ families were planted in plots represented by two rows for each plant, with recommended cultural practices carried out throughout the growing season. The best 30 plants out of the 65 F_3_ generation were chosen based on a selection intensity of 5% for the five selection criteria. In the 2018 season, the selfed seeds of the 30 F_3_ families, as well as the two parents and a commercial variety Giza 86, were planted to represent the F_4_ family. Giza 86 was used as a check variety due to its high yield and good fiber properties. A randomized complete block design with three replications was used; one row represented each plot for each plant, 4.5 m. long and 0.7 m. wide with 70 cm. hill spacing. Hills were thinned to one plant per hill. Normal cultural practices for cotton production were performed at the proper time. The studied characters were the first opening flower, first fruiting node, boll weight (g), seed cotton yield, lint cotton yield, lint percentage, seed index and lint index.

### Statistical and genetic analysis

The PCV and GCV were calculated using the method described by Kearsey and Pooni^39^. The variance and covariance components obtained from the analysis of a regular randomized complete block design were used to estimate the phenotypic and genotypic variances and covariances.

Heritability in a broad sense (h^2^_b_) was calculated as follows:

$${\text{h}}^{{2}} {\text{b }}\left( {{\text{in F}}_{{2}} {\text{generation}}} \right) \, = \, \left( {{\text{VF}}_{{2}} - \left( {{\text{VP}}_{{1}} + {\text{VP}}_{{2}} } \right)/{\text{VF}}_{{2}} } \right) \, \times { 1}00$$

$${\text{h}}^{2} {\text{b }}\left( {{\text{in F}}_{2} {\text{ F}}_{{3{ }}} {\text{and F}}_{4} {\text{ generations}}} \right){ } = { } h_{b}^{2} = \frac{{\delta^{2} g}}{{\delta^{2} ph}} \times 100$$

Heritability in the broad sense was obtained as described by Warner^40^^.^

where VF_2_ is the phenotypic variance of the F_2_ generation, VP_1_ and VP_2_ are the variances of the first and second parents, respectively, σ^2^g is the genotypic variance of the F_3_ and F_4_ generations, and σ^2^ph is the phenotypic variance of the F_3_ and F_4_ generations.

The phenotypic and genotypic correlation coefficients between the studied characters in the F_2_, F_3_, and F_4_ generations were estimated using the method outlined by Dewey and Lu^41^ and Millar et al.^42^.

The appropriate index weights (b, s) were calculated from the following formula. (b) = (P)^−1^⋅(G)⋅(a)^43,44^.

where (b) = Vector of relative index coefficients, (P)^−1^ = Inverse phenotypic variance–covariance matrix, (G) = Genotypic variance–covariance matrix and (a) = Vector of relative economic values based on equally important, i.e., (a)_w_ = (a)_1_ = (a)_2_ = (a)_3_ = 1.

To calculate various selection indices, we used the following formula:

I = b_1_x_1_ + b_2_x_2_ + ⋯ + b_n_x_n_^43,44^.

The formula for estimating the predicted improvement in lint yield based on an index is:

ΔY Selection advance (SA) = SD × Σ(b_i_ × σg_iw_)^1/2^^45^.

where SD is the selection differential in standard units, b_i_ represents the index weights for the characters considered in the index, and σg_iw_ represents the genotypic covariances of the characters with yield.

The formula for estimating the predicted genetic advance in lint yield based on direct selection is ΔG_w_due to selection for X_i_ = K·σg_wi_/σp_i_^46^**.**

The predicted response in any selected and unselected character was calculated based on the methodology proposed by Walker^45^ Robinson and Comstock 48. The realized gains were then determined by computing the deviation of the generation mean for each character from the procedure mean of that character. An ordinary analysis of variance was performed for the randomized complete block design according to Kearsey and Pooni^39^, and estimates were obtained for the phenotypic (PCV %) and genotypic (GCV%) coefficients of variations, phenotypic (σ^2^ph) and genotypic (σ^2^g) variances. The calculation of phenotypic and genotypic correlation coefficients was done using the methodology outlined by Hussain et al.^47^. Heritability in the broad sense was obtained as described by Borojević et al.^48^, and as described by Warner^40^.

### Ethics approval and consent to participate

This article does not contain any studies with human or animal subjects. The current experimental research and field study including the collection of plant material, is complying with relevant institutional, national, and international guidelines and legislation and used for research and development.

## Results

Range, means, phenotypic and genotypic coefficients of variation (PCV and GCV), as well as broad-sense heritability (H^2^b %), for the studied traits across F_2_, F_3_, and F_4_ generations, are presented in Table 1. The results indicate that the mean values for the first opening flower (FOF) were 72.05, 71.98, and 70.54 days, and the PCV values were 4.62%, 3.93%, and 3.19%, while the GCV values were 3.83%, 3.76%, and 3.02%, respectively. Similarly, for the first fruiting node (FFN), the mean values were 7.66, 7.17, and 6.80, and the PCV values were 15.05%, 8.64%, and 6.30%, while the GCV values were 10.78%, 7.78%, and 5.85%, respectively, across F_2_, F_3_, and F_4_ generations. As for boll weight (BW) (g), the mean values were 3.19 g, 3.25 g, and 3.81 g, and the PCV values were 9.88%, 8.20%, and 8.10%, while the GCV values were 7.40%, 7.14%, and 6.63%, respectively, across F_2_, F_3_, and F_4_ generations. Regarding seed cotton yield per plant (SCY/P), the mean values across F_2_, F_3_, and F_4_ generations were 237.01 g, 298.63 g, and 304.21 g/plant, with corresponding PCV values of 35.75%, 17.62%, and 16.36%, and GCV values of 26.85%, 16.16%, and 15.37%. For lint yield per plant (LY/P), the mean values were 88.06 g, 117.15 g, and 126.89 g, with PCV values of 37.15 g, 19.29 g, and 18.58 g, and GCV values of 28.79%, 17.76%, and 17.62%, respectively. As for lint percentage (L%), the mean values were 37.06%, 39.08%, and 41.67%, with PCV values of 5.55%, 5.50%, and 5.19%, and GCV values of 5.26%, 5.42%, and 4.57%. For seed index (SI), the mean values were 11.10 g, 10.29 g, and 12.17 g, with PCV values of 8.13%, 7.38%, and 4.27%, and GCV values of 7.04%, 6.83%, and 4.03%. Concerning the lint index, the mean values were 6.55, 6.63, and 8.73, with PCV values of 11.26%, 11.02%, and 10.46%, and GCV values of 10.26%, 10.00%, and 8.98%. Overall, the results suggest that heritability in broad sense (h^2^b%) values in F_4_ were higher than in F_3_, which were in turn higher than in F_2_ for all the traits analyzed.

**Table 1:** Estimates of broad-sense heritability (h^2^_b_), phenotypic (PCV), and genotypic (GCV) coefficients of variation, range and means, for the eight studied characters in F_2_, F_3,_ and F_4_ generations.

Traits	Generation	Parameters					
		Range		X	PCV (%)	GCV (%)	H^2^_b_
		Min	Max				
FOF	F_2_	63.00	83.00	72.05	4.62	3.83	68.56
	F_3_	67.20	74.00	71.98	3.93	3.76	83.34
	F_4_	65.25	74.25	70.54	3.19	3.02	89.78
FFN	F_2_	4.00	12.00	7.66	15.05	10.78	51.29
	F_3_	5.80	9.20	7.17	8.64	7.78	80.96
	F_4_	5.89	7.56	6.80	6.30	5.85	86.20
BW (g)	F_2_	2.09	4.26	3.19	9.88	7.40	56.08
	F_3_	2.71	3.85	3.25	8.20	7.14	65.28
	F_4_	3.09	4.47	3.81	8.10	6.63	77.68
SCY (g)	F_2_	32.52	545.12	237.01	35.75	26.85	56.43
	F_3_	201.91	434.50	298.63	17.62	16.16	84.15
	F_4_	206.02	389.00	304.21	16.36	15.37	88.34
LY (g)	F_2_	12.08	243.23	88.06	37.15	28.79	60.05
	F_3_	73.46	168.51	117.15	19.29	17.76	84.76
	F_4_	76.98	167.48	126.89	18.58	17.62	89.85
L%	F_2_	31.50	44.62	37.06	5.55	5.26	77.30
	F_3_	33.33	45.15	39.08	5.50	5.42	89.93
	F_4_	36.08	45.54	41.67	5.19	4.57	87.33
SI	F_2_	7.46	13.61	11.10	8.13	7.04	74.90
	F_3_	8.77	12.80	10.29	7.38	6.83	85.67
	F_4_	10.56	13.18	12.17	4.27	4.03	88.96
LI	F_2_	4.26	9.12	6.55	11.26	10.26	66.34
	F_3_	4.80	8.61	6.63	11.02	10.00	83.02
	F_4_	6.38	10.72	8.73	10.46	8.98	91.32

First Opening Flower (FOF), First Fruiting Node (FFN), Boll Weight (BW), Seed Cotton Yield (SCY), Lint Percentage (L%), Seed Index (SI), and Lint Index (LI). The plot uses a smoothed kernel density function to represent the probability of trait values, with the area under the curve indicating the distribution of values and the Y-axis representing the probability.

### Selection criteria

Table 2 presents the mean values, as well as the phenotypic and genotypic coefficients of variation for traits in the five different selection criteria from F_4_ families. The data revealed that selection criterion I1 had the highest value for the first opening flower at 67.14, as well as the highest PCV and GCV values for the first fruiting node at 7.33% and 6.74%, respectively. Selection criterion I2 yielded the highest mean value for both seed cotton yield (122.93) and seed index (12.58). Additionally, selection criterion I3 had the highest PCV and GCV values for the first opening flower, boll weight, seed index, and lint index, with values of 2.48%, 2.18%, 11.11%, 10.39%, 5.86%, 5.43%, and 13.59%, 13.33%, respectively. Selection criterion I4 showed the highest mean values for boll weight, lint yield, lint percentage, and lint index, with values of 4.11, 51.94, 42.60, and 9.27, respectively. Finally, selection criterion I5 gave the best mean value for the first fruiting node at 6.66, and the highest values for PCV and GCV for seed cotton yield and lint yield with values of 19.07%, 18.22%, and 21.81%, 20.95%, respectively. The highest PCV and GCV estimates suggest that the first fruiting node, seed cotton yield, boll weight, first flower opening, seed index, lint yield, and lint percentage are mainly under genetic control and less affected by the environment.

**Table 2:** Mean, phenotypic (PCV) and genotypic coefficient of variations (GCV) for earliness, yield, and its components for the five different selection criteria parameters of F_4_ selected families.

Selection criteria	Parameters	Traits							
		FOF	FFN	BW (g)	SCY (g)	LY (g)	L%	SI	LI
I1	Mean	67.14	6.71	3.69	111.34	43.87	39.50	12.28	8.03
	PCV	2.02	7.33	6.14	17.63	15.96	3.87	3.39	5.66
	GCV	1.90	6.74	4.91	17.17	15.51	3.80	3.05	5.30
I2	Mean	71.00	6.73	4.02	122.93	51.31	41.55	12.58	8.98
	PCV	1.36	6.73	7.61	16.91	19.78	6.32	3.74	9.74
	GCV	0.95	6.44	6.80	15.92	18.79	6.25	3.55	9.20
I3	Mean	71.50	7.17	3.73	119.91	47.39	39.40	12.07	7.90
	PCV	2.48	4.25	11.11	13.42	16.78	5.65	5.86	13.59
	GCV	2.18	3.33	10.39	11.91	15.53	5.63	5.43	13.33
I4	Mean	71.23	6.76	4.11	121.73	51.94	42.60	12.47	9.27
	PCV	1.98	5.23	5.35	16.16	16.89	2.63	3.55	5.85
	GCV	1.61	4.90	4.10	15.17	15.82	2.45	3.34	5.04
I5	Mean	70.92	6.66	4.08	118.51	50.02	41.99	12.52	9.08
	PCV	1.19	5.85	5.91	19.07	21.81	5.64	4.06	8.32
	GCV	0.64	5.59	4.88	18.22	20.95	5.57	3.88	7.72

First Opening Flower (FOF), First Fruiting Node (FFN), Boll Weight (BW), Seed Cotton Yield (SCY), Lint Percentage (L%), Seed Index (SI), and Lint Index (LI). The plot uses a smoothed kernel density function to represent the probability of trait values, with the area under the curve indicating the distribution of values and the Y-axis representing the probability.

### Mean performance of the thirty selected families of F_4_ generation

Table 3 displays the average performance of thirty selected families from the F_4_ generation of the Giza 94X Suvin population for all traits studied. Significant differences were noted among various progenies compared to the better parent and commercial variety. For the first opening flower trait, progenies No. 11, 14, and 21 showed significant differences from the superior parent, while progenies No. 12 and 24 exhibited highly significant differences. When compared to the commercial variety, progenies No. 2, 18, 27, and 29 differed significantly, and progenies No. 11, 12, 13, 14, 15, 21, 24, and 25 differed highly significantly. In terms of the first fruiting node trait, progenies No. 11 and 12 differed significantly from the superior parent, while progeny No. 2 showed highly significant differences. Progenies No. 3, 5, 10, 17, and 20 also exhibited significantprogenies No. 1, 2, 6, 11, 12, 14, 18, 24 , 27, and 30 differing significantly compared to the commercial variety. For boll weight, progenies No. 16 and 29 significantly and highly significantly differed from the superior parent, respectively. Progenies No. 3 and 7 showed significant differences and progenies No. 6, 16, and 29 differed significantly compared to the commercial variety. The seed cotton yield ranged from 90.85 g (progeny No. 1) to 167.06 g (progeny No. 20). Progenies No. 6, 17, and 22 significantly and highly significantly differed from the superior parent, respectively. Progenies No. 17, 22, and 23 also shwoed significant and highly significant differences from the commercial variety, respectively. For lint yield, progenies No. 6, 9, 13, 17, 19, 20, and 28 highly differed from the better parent, with progenies No. 21, 22, and 29 showing significant differences. Progenies No. 6, 9, 13, 17, 19, 20, and 28 also differed highly significantly from the commercial variety, with progenies No. 21, 22, and 29 showing significant differences. Regarding the lint percentage trait, progenies No. 25 and 27 exhibited significant and highly significant differences from the better parent, respectively. Meanwhile, twenty families significantly differed from the commercial variety. For the seed index trait, progenies No. 1, 2, 6, 13, 16, and 29 showed highly significant differences from the superior parent. Progenies No. 14, 26, and 28 exhibited significant and highly significant differences from the commercial variety, respectivelyIn terms of the lint index trait, progenies No. 9, 15, and 20 significantly and highly significantly differed from the superior parent, respectively. Progenies No. 6 , 7, 16, 17, 18, 19, 28, 29, and 30 also showed significant and highly significant differences from the superior parent, respectively. Progenies No. 4, 5, 13, 24, and 27 exhibited significantly and highly significantly differed from the commercial variety, respectively.

**Table 3:** Mean performance of thirty selected families from the F_4_ generation of the Giza 94X Suvin population for all studied traits.

Families	FOF	FFN	BW (g)	SCY (g)	LY (g)	L%	SI	LI
1	71.00	6.44	3.87	90.85	34.13	37.55	13.04	7.84
2	70.00	5.89	4.12	101.11	37.39	36.99	13.17	7.74
3	72.33	6.67	4.18	110.36	45.99	41.67	12.06	8.62
4	71.00	7.00	3.74	91.46	38.13	41.68	11.81	8.44
5	71.00	6.67	3.98	101.56	41.85	41.20	11.94	8.37
6	70.33	6.44	4.32	131.89	56.38	42.74	12.97	9.68
7	74.00	6.89	4.15	96.99	40.54	41.84	12.70	9.14
8	74.00	7.56	3.09	113.96	40.71	35.69	10.73	5.96
9	70.67	7.22	4.12	141.17	59.53	42.16	12.24	8.92
10	71.00	6.67	3.84	94.02	36.84	39.18	12.26	7.90
11	67.00	6.22	3.63	98.39	40.01	40.67	11.88	8.15
12	65.00	6.22	3.52	93.78	36.29	38.68	11.85	7.48
13	69.00	7.56	3.66	142.55	56.34	39.49	12.93	8.44
14	67.00	6.44	4.05	97.77	38.55	39.42	12.30	8.00
15	69.00	7.00	4.09	115.47	47.55	41.19	12.58	8.81
16	71.00	7.22	4.50	113.93	48.34	42.45	13.01	9.61
17	71.33	6.67	3.88	129.62	57.32	44.16	12.08	9.57
18	69.67	6.33	4.00	109.96	48.64	44.17	12.12	9.60
19	72.33	7.22	4.03	147.25	63.68	43.22	12.53	9.55
20	71.00	6.67	3.99	167.06	70.54	42.25	12.13	8.88
21	67.00	7.00	3.37	135.48	49.60	36.60	12.74	7.36
22	71.33	7.22	3.86	129.00	50.44	39.08	12.67	8.14
23	73.00	7.33	3.37	125.83	49.24	39.09	11.94	7.67
24	66.33	6.56	3.91	109.67	44.54	40.61	12.03	8.24
25	68.67	7.00	3.72	101.71	41.73	41.03	12.26	8.53
26	74.00	7.44	3.99	110.17	45.37	41.16	12.43	8.71
27	70.00	6.56	3.88	113.49	46.51	40.90	11.91	8.26
28	71.00	6.89	4.09	133.48	58.05	43.45	12.34	9.50
29	70.00	6.33	4.55	119.82	50.94	42.47	13.33	9.84
30	70.33	6.56	4.00	107.22	47.03	43.76	12.57	9.79
overall mean	70.31	6.80	3.92	115.83	47.41	40.82	12.35	8.56
better parent	69.67	6.67	3.96	80.29	31.90	39.72	13.25	8.73
commercial variety	72.33	7.21	3.73	106.53	41.46	38.86	11.78	7.48
L.S.D 0.05	2.03	0.45	0.42	18.31	7.95	1.04	0.50	0.74
L.S.D 0.01	2.70	0.60	0.56	24.37	10.57	1.38	0.66	0.99

First Opening Flower (FOF), First Fruiting Node (FFN), Boll Weight (BW), Seed Cotton Yield (SCY), Lint Percentage (L%), Seed Index (SI), and Lint Index (LI). The plot utilizes a smoothed kernel density function to show the probability of trait values. The area under the curve indicates the distribution of values with the Y-axis representing the probability.

### Phenotypic and genotypic correlation

Phenotypic and genotypic correlation coefficients were calculated for all studied traits in the F_4_ selected families (Table 4). Positive and highly significant correlation values wereobserved for first opening flower and first fruiting node (0.445* and 0.521**), first fruiting node vs seed index (0.410* and 0.591**), seed cotton yield with both lint yield and seed index (0.848** and 1.000**) and (0.809** and 0.912**) respectively. Additionally, the correlation values between boll weight with bothlint percentage and lint index were also highly significant and positive (0.560** and 0.664**) and (0.685** and 0.851**), respectively. Conversely,a negative correlation was observed between boll weight (-0.349*) and seed index (-0.472**). Highly significant positive correlation values were observed between seed cotton yield and both lint yield and seed index (0.848** and 1.000**) and (0.809** and 0.912**). Furthermore, for lint yield, positive highly significant correlation values (0.503** and 0.532**), (0.725** and 0.747**) and (0.516** and 0.531**) were observed between lint yield and both lint percentage, seed index and lint index (0.503** and 0.532**), (0.725** and 0.747**) and (0.516** and 0.531**).

**Table 4:** Coefficient of phenotypic and genotypic correlations among different character combinations of 8 quantitative traits for the F_4_ selected families.

Traits	FOF	FFN	BW (g)	SCY (g)	LY (g)	L%	SI	LI
FOF		0.445*	0.063	0.130	0.120	0.121	0.071	0.091
FFN	0.521**		− 0.227	0.325	0.210	− 0.075	0.410*	− 0.130
BW(g)	0.149	− 0.348		0.056	0.215	0.560**	− 0.349*	0.685**
SCY(g)	0.178	0.442*	0.069		0.848**	0.234	0.809**	0.256
LY(g)	0.236	0.383*	0.273	1.000**		0.503**	0.725**	0.516**
L%	0.173	− 0.075	0.664**	0.261	0.532**		− 0.090	0.906**
S.I	0.129	0.591**	− 0.472**	0.912**	0.747**	− 0.098		− 0.110
L.I	0.152	− 0.112	0.851**	0.297	0.531**	0.938**	− 0.149	

*and ** significant and highly significant at 0.05 and 0.01 probability levels, respectively. First Opening Flower (FOF), First Fruiting Node (FFN), Boll Weight (BW), Seed Cotton Yield (SCY), Lint Percentage (L%), Seed Index (SI), and Lint Index (LI). The plot uses a smoothed kernel density function to represent the probability of trait values, with the area under the curve indicating the distribution of values and the Y-axis representing the probability.

### Density plots for the studied traits in the three generations

**Figure 1:**
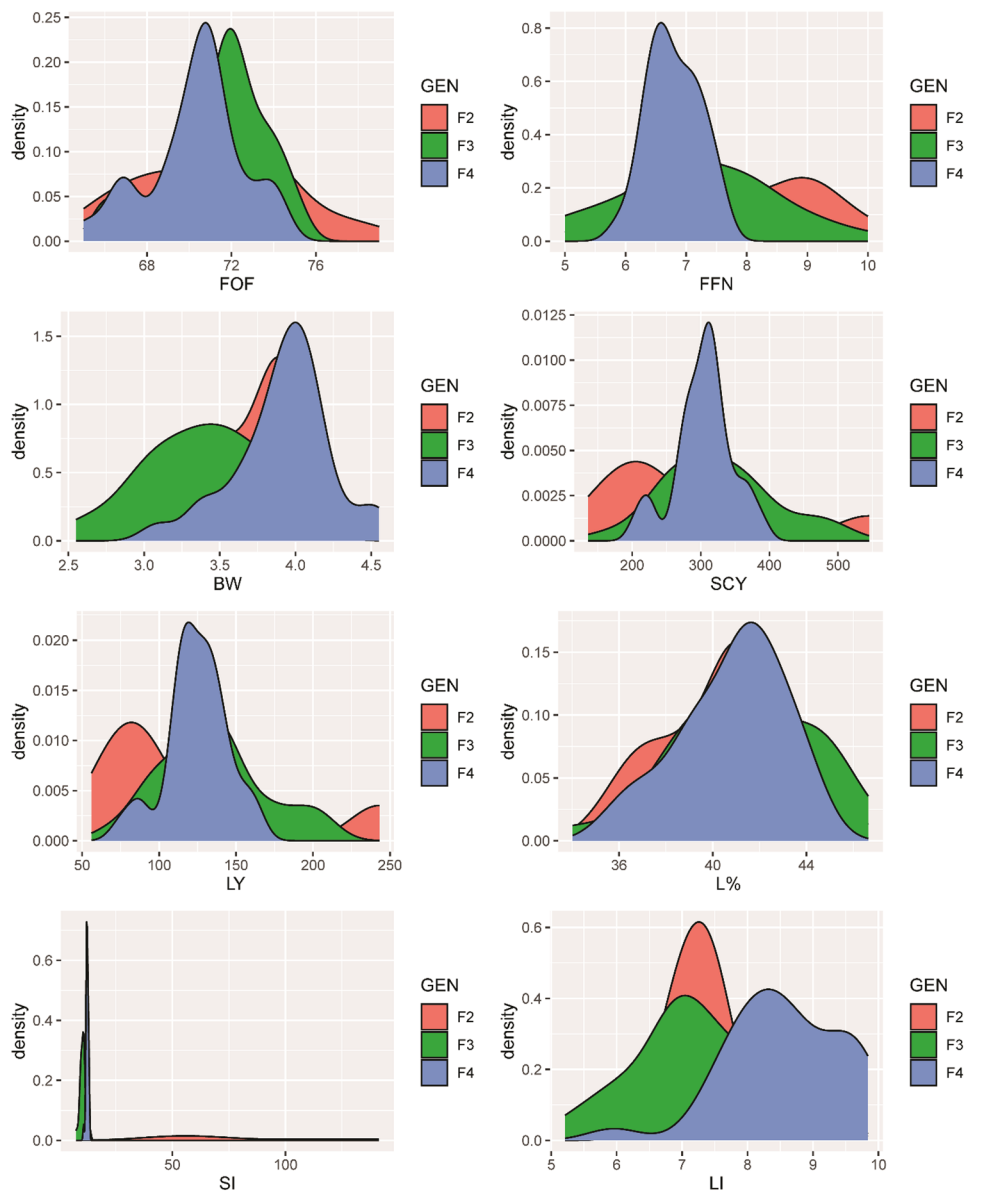
The density plot of six studied traits: First Opening Flower (FOF), First Fruiting Node (FFN), Boll Weight (BW), Seed Cotton Yield (SCY), Lint Percentage (L%), Seed Index (SI), and Lint Index (LI). The plot uses a smoothed kernel density function to represent the probability of trait values, with the area under the curve indicating the distribution of values and the Y-axis representing the probability.

Figure 1 displays density plots displays the studied traits in the three generations using a smoothed kernel density function to represent the probability of the trait values. The area under the curve represents the distribution of values, and the Y-axis represents their probability. The X-axis value corresponding to the peak of each density plot is the average of the trait. The peaks of each density plot show a higher concentration of values and therefore a higher probability, while the tails demonstrate a lower concentration of values and a lower probability. The figure indicates that the peak value of LI for F_2_ is different from F_3_ and F_4_, with the means higher than the mean of LI for F_3_ and F_4_. A similar trend is observed for SI, while the opposite is true for other traits. The density plots demonstrate that selection from F_2_ improved the means of LI and SI but decreased the variation of all studied traits.

### Principal component analysis (PCA)

**Figure 2:**
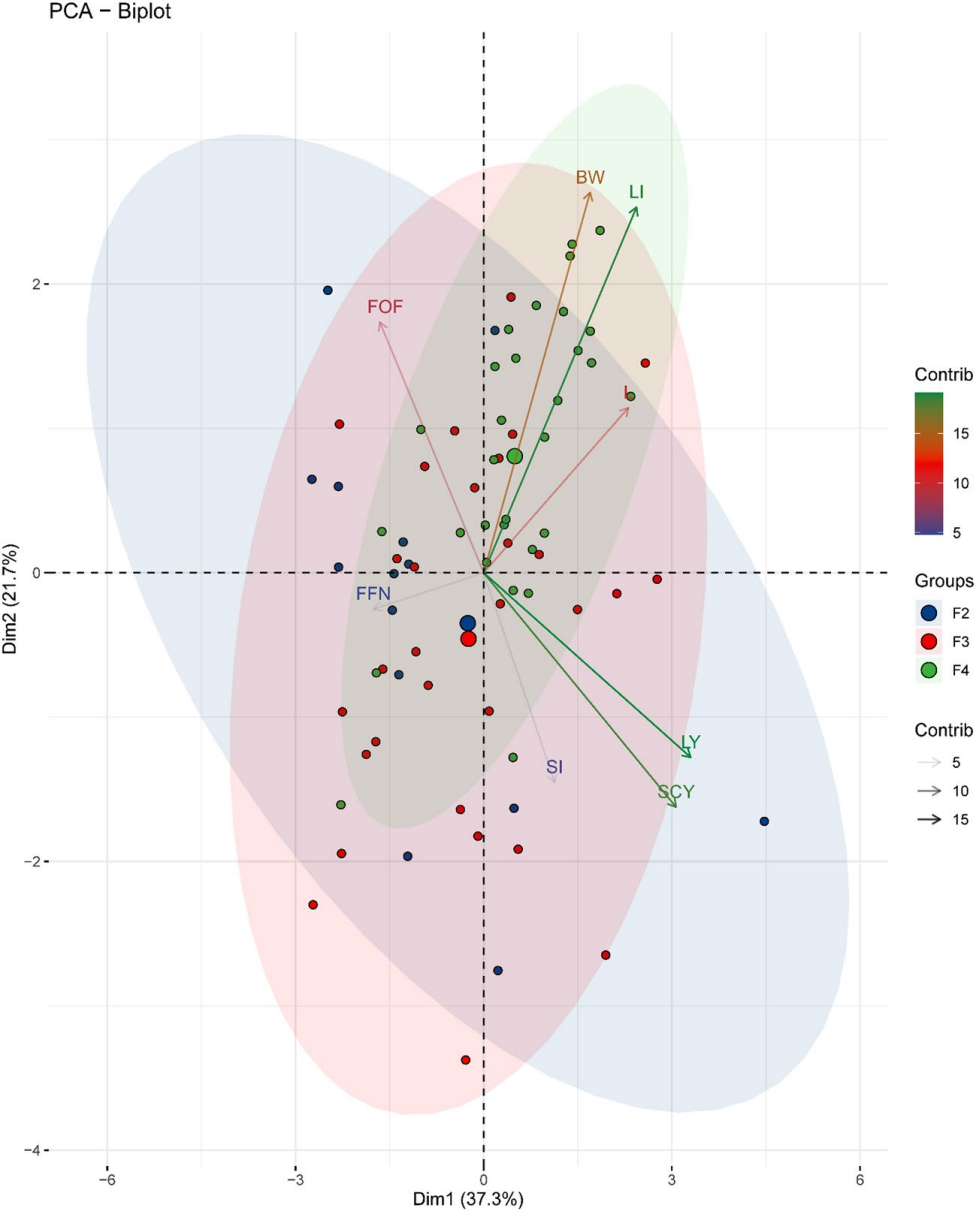
The PCA biplot for the three generations and traits (with a sample size of 8) displays the contribution of individuals to each variable. Individuals on the same side as a variable are seen as having a high impact on it. The strength of contribution to each principal component (PC) is shown by the magnitude of the vectors (lines). The direction of the vectors indicates the correlation between variables, with vectors pointing in similar directions showing positive correlation, vectors pointing in opposite directions indicating negative correlation, and vectors at proximately right angles indicating low or no correlation. The colored concentration ellipses represent the observations grouped by mark class, with the size determined by a 0.95 probability level.

In biplot analysis (Fig. 2), the sharp angle (below 90 degrees) and the obtuse angle (above 90 degrees) between the variables indicated positive and negative correlation between variables, respectively. Positive correlations were observed among the indices of BW, LI, L, LY, SI, and SCY. These indices were highly positively correlated with F_2_, F_3_ and F_4_.

### Path analysis

**Figure 3:**
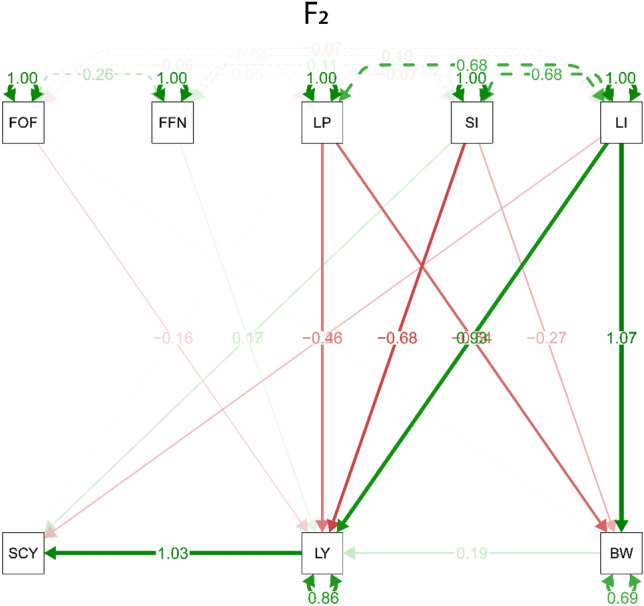
Path diagram that illustrates both the direct and indirect effects of the studied traits in the F_2_ generation. Bidirectional arrows show correlation between the variables, and unidirectional arrows indicate a direct effect on the direction of the arrow, blue and red arrows represent positive and negative effects. Solid arrows indicate *P* < 0.05 and dashed arrows indicate *P* > 0.05. First Opening Flower (FOF), First Fruiting Node (FFN), Boll Weight (BW), Seed Cotton Yield (SCY), Lint Percentage (L%), Seed Index (SI), and Lint Index (LI). The plot uses a smoothed kernel density function to represent the probability of trait values, with the area under the curve indicating the distribution of values and the Y-axis representing the probability.

**Figure 4:**
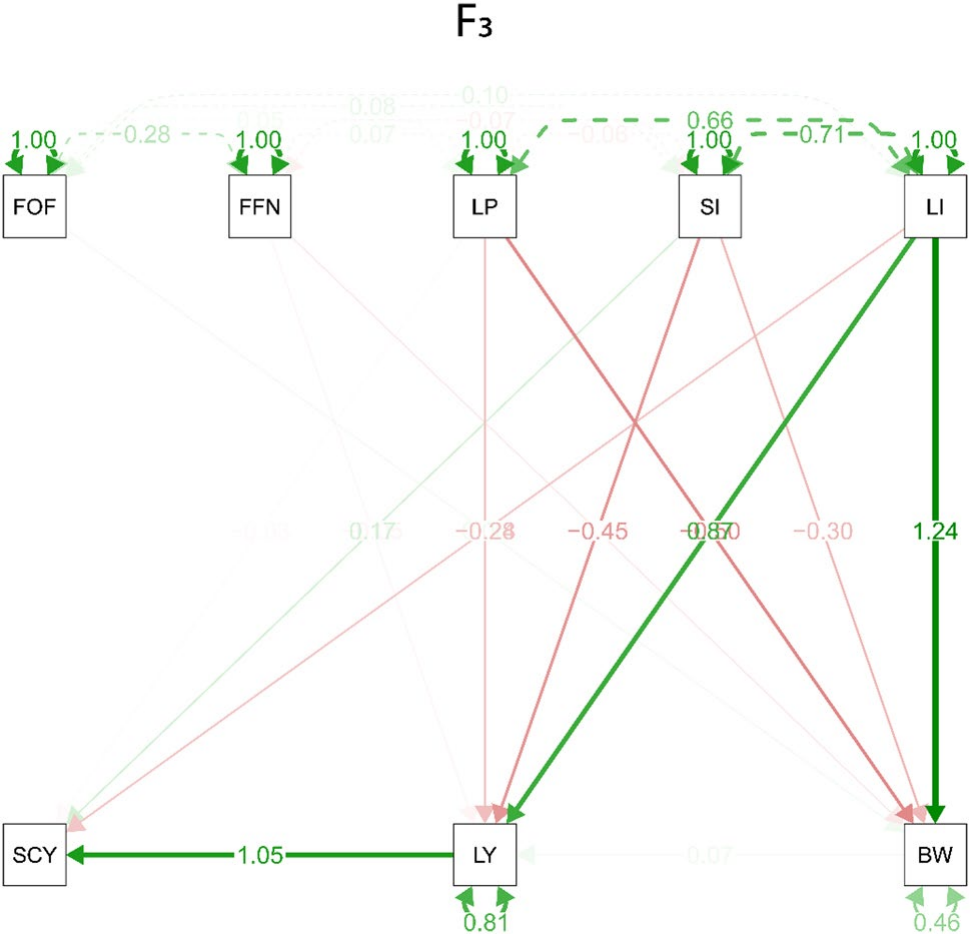
Path diagram that illustrates both the direct and indirect effects of the studied traits in the F_2_ generation. Bidirectional arrows show correlation between the variables, and unidirectional arrows indicate a direct effect on the direction of the arrow, blue and red arrows represent positive and negative effects. Solid arrows indicate *P* < 0.05 and dashed arrows indicate *P* > 0.05. First Opening Flower (FOF), First Fruiting Node (FFN), Boll Weight (BW), Seed Cotton Yield (SCY), Lint Percentage (L%), Seed Index (SI), and Lint Index (LI). The plot uses a smoothed kernel density function to represent the probability of trait values, with the area under the curve indicating the distribution of values and the Y-axis representing the probability.

**Figure 5:**
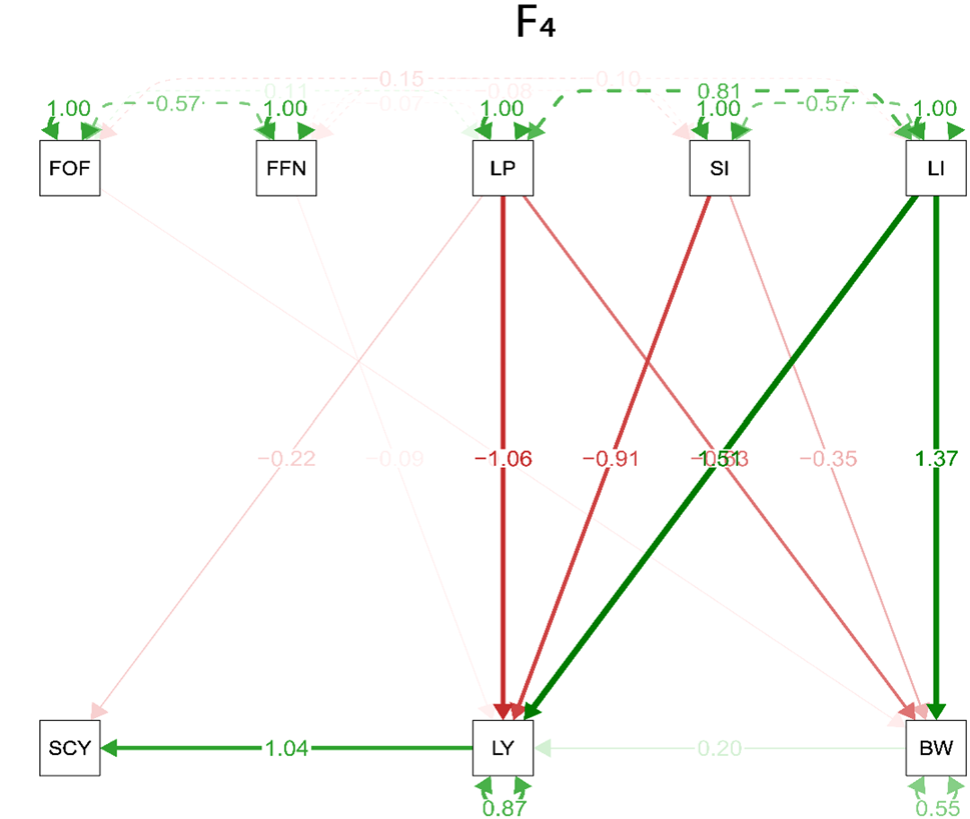
Path diagram that illustrates both the direct and indirect effects of the studied traits in the F_2_ generation. Bidirectional arrows show correlation between the variables, and unidirectional arrows indicate a direct effect on the direction of the arrow, blue and red arrows represent positive and negative effects. Solid arrows indicate *P* < 0.05 and dashed arrows indicate *P* > 0.05. First Opening Flower (FOF), First Fruiting Node (FFN), Boll Weight (BW), Seed Cotton Yield (SCY), Lint Percentage (L%), Seed Index (SI), and Lint Index (LI). The plot uses a smoothed kernel density function to represent the probability of trait values, with the area under the curve indicating the distribution of values and the Y-axis representing the probability.

The study utilized path analysis, a statistical method for examining causal relationships between variables, using the "sem" function in the "lavaan" package of R software. The results were displayed in path diagrams (Figs. 3, 4 and 5) that illustrated the effects of five factors (lint index, seed index, lint percentage, first opening flower, and first fruiting nod) on three outcomes (boll weight, lint yield, and seed cotton yield) across three generations. Three types of arrows were used in the path diagrams: single-headed arrows indicating causal relationships, double-headed arrows indicating covariance between two factors, and double-headed arrows pointing to a single factor representing the variance of that factor. The findings revealed that the lint index had a strong positive effect on both lint yield and boll weight in all three generations, with R^2^ values of 0.93, 0.87, and 1.51 for lint yield and 1.07, 1.24, and 1.7 for boll weight in F_2_, F_3_, and F_4_, respectively. Moreover, lint yield had a direct and significant effect on seed cotton yield in all three generations, with R^2^ values of 1.03, 1.05, and 1.04 for F_2_, F_3_, and F_4_, respectively. Boll weight had a stronger direct effect on lint yield than both lint percentage and boll weight themselves, as evidenced by a more substantial direct effect of boll weight on lint yield than the direct effect of lint percentage and boll weight on lint yield. The indirect effect of the lint index and seed index on lint yield through lint percentage had the same pattern across all three generations, where the lint index had a positive effect and the seed index had a negative effect. Meanwhile, the seed index had no indirect effect on lint yield through boll weight, whereas the lint index and seed index had a positive indirect effect on lint yield through boll weight in all three generations. Finally, the direct and indirect effects on lint yield were more pronounced in F_4_ than in F_2_ and F_3_, possibly due to selection that strengthened the relationship between lint yield and its components, resulting in a more stable progeny of that generation. In summary, the path analysis provided valuable insights into the causal relationships among different factors and their effects on lint yield, boll weight, and seed cotton yield across three generations.

## Discussion

The results indicate that genetic factors play a significant role and that there is a possibility of achieving positive outcomes by selecting early segregating generations in the presence of water deficit stress. These findings are consistent with earlier research^32,49,50^. The F_2_ and F_3_ generations showed greater phenotypic and genotypic coefficients of variation for all traits studied compared to the F_4_ generation. This decrease in genetic variability and heterozygosity may have resulted from various selection methods that reduced a significant portion of the variability^30,51,52^. Although the mean performance of all traits studied was higher in the F4 generation than in the F_3_ generation, the favorable alleles that accumulated due to effective selection procedures resulted in a lower desirable MR in the F_4_ generation. The study suggests that selecting for these traits is possible, and other researchers have reported similar results^5,49,50,53^. Plant breeders need to consider all the economic traits and not just focus on one. Correlation analysis is a useful tool for predicting how a change in one trait will affect another. The genotypic correlations were generally higher than the phenotypic correlations, which could be attributed to the relative stability of genotypes under certain selection pressures. This finding is consistent with other studies^16,37,43,51,54^. The study showed that most traits exhibited an increase in mean values across generations in the population due to additive gene action. Additionally, high heritability values were observed, possibly due to the close relationship between PCV and GCV values. All traits under study showed a very high to high degree of heritability, indicating a low or negligible influence of the environment on their expression, making them suitable for selection and improvement. The findings are in agreement with Ali et al.'s research^53^ which reported a persistent correlation between GCV and PCV values, leading to high estimates of broad-sense heritability. The results also indicated a minor disparity between GCV and PCV values for most traits, suggesting that environmental factors have a minimal impact on their expression. The variation in the values of genotypic and phenotypic coefficients was narrow for all studied characters which indicated less influence of environment in the expression of these characters^35^. The high values of PCV and GCV together with high values of heritability are a good indicator for genetic advance in this population through the amount of genetic variance to be expected from the selection. It was suggested that combining the genetic coefficient of variation with heritability would provide the most accurate estimation of the expected genetic variance resulting from selection^8,14^. The three most influential factors in genetic progress in the three populations were high selection intensity, genotypic coefficient of variation, and heritability^1,10,32,37,43,53,55^. The differences between generations may be due to the various genotypes scored by each selection criteria. The study found that selection criteria I2, I4, and I5 were effective in improving most yield and its component traits, while criterion I1 was efficient in improving earliness traits. The narrow range between PCV and GCV values for different selection criteria suggests that environmental factors have a minimal impact on these traits. The analysis of variance revealed significant differences among families of F_4_ for all studied traits, indicating that selection criteria would be effective. The increase in selected families was greater than the better parent and commercial variety. The results also showed variations in the mean performance of most selected families, which could be attributed to gene expression. Therefore, selecting adaptable families with higher means than the better parent and commercial variety would help produce highly yielding and earlier crops, particularly in winter cropping, without a decrease in yield^23,27,31,39,56^. It appeared that most of the yield and its component traits were positive and either significant or highly significant correlated with each other, indicating that these traits are important components of cotton yield. A positive association between major yield components is very significant to the breeder because component breeding would be very effective under such a situation. Such correlations indicate that selection for improving one or more of these traits would improve other traits^17,36,57^. The increases in yield and its components for this population were higher in family numbers (9, 13, 19, 20 and 21). These families could be continued to further generations as breeding genotypes for developing higher yield and its components. Nazmey et al.^58^ studied cotton yield and yield components in relation to the relative contribution and found that seed cotton yield was significantly and positively correlated with boll weight and lint yield. The same relationship was found between lint yield and each boll weight and seed cotton yield^59–61^.Which reported that positive significant correlation values were found between seed cotton and lint yields with boll weight and lint percentage. It was indicated that seed cotton yield was highly significantly genetically correlated with boll weight (r = 0.99), lint yield (r = 0.88) and lint index (r = 0.96). With respect to, the phenotypic and genotypic correlation values between lint percentage and lint index there were positive highly significant correlation values (0.906** and 0.938**)^62^.

## Conclusion

Based on our findings, it can be concluded that the studied cotton traits, including first opening flower, first fruiting node, boll weight, seed cotton yield, lint yield, lint percentage, seed index, and lint index, are significantly influenced by genetic factors. The broad-sense heritability values indicate that the traits are heritable, with higher heritability values observed in the F_4_ generation compared to F_3_ and F_2_. The results of the different selection criteria indicate that the traits under genetic control are suitable for breeding programs. The mean performance of selected families from the F_4_ generation of the Giza 94X Suvin population showed significant differences in various progenies compared to the better parent and commercial variety, indicating the potential for further selection and improvement of cotton traits. There are several prospects for future research and breeding programs aimed at improving cotton traits. Firstly, the traits found to be under genetic control and suitable for breeding programs, including first fruiting node, seed cotton yield, boll weight, first flower opening, seed index, lint yield, and lint percentage, can be further studied to identify specific genetic markers and genes responsible for these traits. This information can then be used to develop more efficient breeding strategies, such as marker-assisted selection, to improve these traits in cotton varieties. Secondly, the selected families from the F_4_ generation with significant differences in progenies compared to the better parent and commercial variety can be further evaluated for their agronomic performance and adaptability to different growing conditions. This information can then be used to identify the best-performing families and varieties, which can be released for commercial cultivation. Lastly, the heritability values observed in the F_4_ generation suggest that this generation may be suitable for developing new cotton varieties through the application of different breeding methods, such as recurrent selection or hybridization with other elite varieties. The resulting new varieties can then be evaluated for their performance and suitability for cultivation in different regions.

## Data Availability

All data generated or analyzed during this study are included in this article.
